# Underestimation of carotid plaque by ultrasound IMT and potential error in measurement of change in plaque burden: simultaneous comparison with 3 T MRI

**DOI:** 10.1186/1532-429X-11-S1-P171

**Published:** 2009-01-28

**Authors:** Mark Bowers, Leanne Harmann, Robert Prost, Megan Bright, Anil Doppalapudi, Tayyab Mohyuddin, John LaDisa, Raymond A Migrino

**Affiliations:** 1 Medical College of Wisconsin, Milwaukee, WI USA; 2 Marquette University, Milwaukee, WI USA

**Keywords:** Carotid Plaque, Plaque Burden, Plaque Volume, Posterior Wall Thickness, Flow Divider

## Introduction

Both ultrasound (intima-media thickness, IMT) and MRI are established methods of measuring carotid plaque burden. The recent ENHANCE trial demonstrated that despite greater reduction in LDL cholesterol with simvastatin-ezetimibe, carotid IMT did not decrease and in fact trended towards progression versus simvastatin control. This was a surprising finding and called into question whether measuring the change in posterior wall thickness by 2-dimensional IMT accurately reflects the change in plaque burden especially since plaque can be eccentric. Unlike IMT that by standard methodology quantifies posterior wall thickness, MRI allows measurement of plaque volume and is ideal for quantifying eccentric plaques.

## Purpose

To compare carotid plaque volume calculated from ultrasound IMT and lumen radius (IMT-vol) versus plaque volume measured by MRI (MRI-vol) in patients with atherosclerotic disease and to compare % change in IMT versus MRI-vol in patients taking statins for 6 months.

## Methods

16 patients (67 ± 10 years, 3 females) with carotid atherosclerotic disease underwent sequential B-mode ultrasound and MRI of both carotid arteries at baseline, with 11 having 6-month follow up post-statin treatment. IMT of the right and left common carotid (CC, starting 2–4 mm below flow divider and 10 mm length going inferiorly) and internal carotid (IC, starting 2 mm above flow divider and 10 mm length going superiorly) arteries were measured from the posterior wall using standard ENHANCE trial methods. IMT-vol was calculated using the formula: [π(r+IMT)^2^-πr^2^]*10 mm where r is the lumen radius and IMT is the mean posterior plaque thickness. MRI-vol was derived from T2-weighted spin echo images using 3 T MRI (General Electric) and 4 channel carotid coil (0.47 × 0.47 × 2 mm spatial resolution, 256 × 256 matrix). Plaque volume in 10 mm length of the corresponding CC and IC regions were derived using Simpson's formula from sequential 2 mm slice thickness images. IMT-vol and MRI-vol were compared in 61 evaluable carotid segments and % change in volume was compared in subjects with 6 month follow-up.

## Results

**Figure 1:**
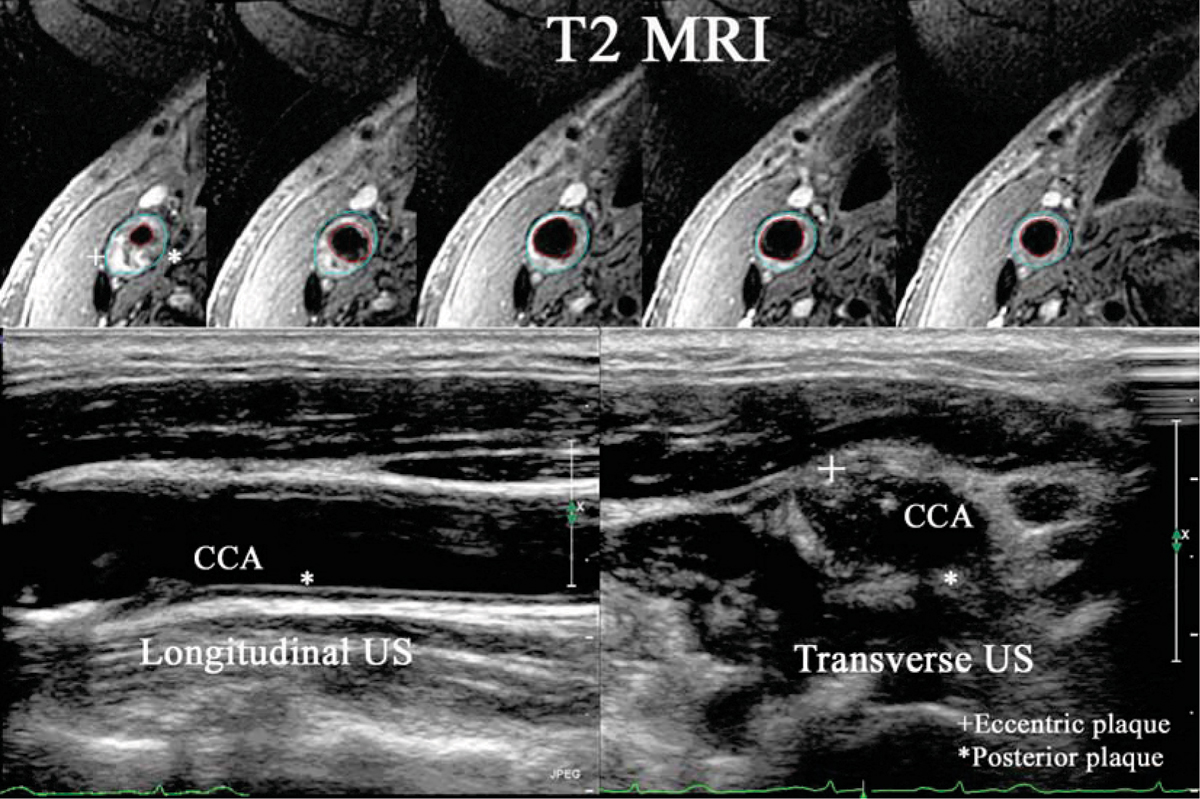
Figure 1

Carotid plaques often have eccentric distribution (see Figure 1). IMT-vol has significant correlation with MRI-vol (R = 0.5, p < 0.001). However, IMT-vol is significantly less than MRI-vol (all, CC, IC: 227 ± 159 vs. 430 ± 159; 211 ± 63 vs. 507 ± 120; 232 ± 211 vs. 405 ± 164 mm^3^, all p < 0.001). Following 6-month statin treatment, IMT increased by 2.1 ± 36%, IMT-volume increased by 15 ± 61% while MRI-vol decreased by 9.4 ± 15%. The % change in MRI-vol did not correlate with % change in IMT (R = 0.2, p = 0.3) or IMT-vol (R = 0.1, p = 0.6). When % change is categorized into 3 groups (greater than +5%, +5 to -5% or greater than -5%), IMT-vol was concordant with MRI-vol change in only 8/21 (38%) of cases.

## Conclusion

Carotid plaque burden derived from posterior wall IMT systematically underestimates plaque volume as measured by MRI, likely due to eccentric nature of carotid atherosclerosis. The change in plaque volume measured by MRI is discordant with change in IMT or IMT-vol suggesting non-uniform change in regional plaque burden that may not be reflected by simply measuring the posterior wall. This has important implications in utilization of ultrasound in serial studies of plaque burden and suggests need for 3-dimensional volumetric measurement of plaque burden.

